# Image discrimination reversal learning is impaired by sleep deprivation in rats: Cognitive rigidity or fatigue?

**DOI:** 10.3389/fnsys.2022.1052441

**Published:** 2022-11-17

**Authors:** Brian K. Strobel, Michelle A. Schmidt, Daniel O. Harvey, Christopher J. Davis

**Affiliations:** Department of Translational Medicine and Physiology, Sleep and Performance Research Center, Elson S. Floyd College of Medicine, Steve Gleason Institute for Neuroscience, Washington State University, Spokane, WA, United States

**Keywords:** pairwise discrimination, cognitive flexibility, operant conditioning, adaptive decision feedback, perseverative errors, response latency

## Abstract

**Introduction:**

Insufficient sleep is pervasive worldwide, and its toll on health and safety is recapitulated in many settings. It is thus important to understand how poor sleep affects the brain and decision making. A robust literature documents the adverse effects of sleep deprivation on cognitive processes including cognitive flexibility, which is the capacity to appraise new feedback and make behavioral adjustments to respond appropriately. Animal models are often used to unravel the molecules, genes and neural circuits that are altered by sleep loss. Herein we take a translational approach to model the effects of sleep deprivation on cognitive rigidity, i.e., impaired cognitive flexibility in rats.

**Methods:**

There are several approaches to assess cognitive rigidity; in the present study, we employ a pairwise discrimination reversal task. To our knowledge this is the first time this paradigm has been used to investigate sleep deprivation. In this touchscreen operant platform, we trained rats to select one of two images to claim a sucrose pellet reward. If the non-rewarded image was selected the rats proceeded to a correction trial where both images were presented in the same position as before. This image presentation continued until the rat selected the correct image. Once rats reached performance criteria, the reward contingencies were reversed. In one group of rats the initial reversal session was preceded by 10 h of sleep deprivation. We compared those rats to controls with undisturbed sleep on the number of sessions to reach performance criteria, number of trials per session, response latencies, correct responses, errors, perseverative errors and perseveration bouts in the initial training and reversal phases.

**Results:**

We report that on reversal session one, sleep deprived rats completed a fraction of the trials completed by controls. On subsequent reversal sessions, the sleep deprived rats struggled to adapt to the reversed contingencies despite completing a similar number of trials, suggesting an effect of cognitive rigidity separate from fatigue.

**Discussion:**

We discuss the delayed performance dynamics incurred by sleep loss in the context of fatigue and the implications of using pairwise discrimination reversal as a model to further examine the effects of sleep loss on adaptive decision making.

## Introduction

Insufficient sleep is an increasingly common issue worldwide (Banks and Dinges, 2007; Roenneberg, 2013; Hafner et al., 2017). There are many causes of sleep loss in stressful and fast-paced societies, including our society’s dependence on shift work (Ganesan et al., 2019). The adverse effects of insufficient sleep are evident across a variety of settings and can result in workplace hazards, unsafe roads, illness, and long-term health consequences (Hafner et al., 2017; James et al., 2017; Ganesan et al., 2019; McHill and Wright, 2019). Understanding the specific effects sleep loss has on performance—especially cognition—is critical for developing mitigation strategies to limit its effect in demanding environments such as military operations, medical care, and emergency responses.

Exactly how sleep loss affects cognition is complex and not fully understood, in part because cognition is multifaceted. Moreover, sleep deprivation (SD) varies in type, duration and frequency further complicating our attempts to draw definitive conclusions. Despite these difficulties a sustained and active area of research is directed at elucidating the distinct effects of SD on different aspects of cognition. There is a large literature demonstrating evidence that sleep loss incurs temporary deficits in vigilance (measured by the psychomotor vigilance test) (Banks and Dinges, 2007; Killgore, 2010; Kayser et al., 2022), attention (Goel et al., 2009; Killgore, 2010; Kayser et al., 2022), emotional functioning (Killgore, 2010; Kayser et al., 2022), mood (Killgore, 2010; Kayser et al., 2022), and learning (Goel et al., 2009; Killgore, 2010). There is also evidence of the effects of insufficient sleep on sensory perception (Killgore, 2010; Zhou et al., 2022), appraisal (Yoo et al., 2007a; Killgore, 2010) and decision making (Killgore, 2010; Kayser et al., 2022). The effects of SD on more executive processes are not as clear and involve different areas of the brain. Despite SD having an apparent effect on the prefrontal cortex on brain imaging studies (Harrison and Horne, 2000; Harrison et al., 2000), some aspects of executive functioning are preserved after SD (Harrison and Horne, 2000; Lim and Dinges, 2010; Tucker et al., 2010).

Cognitive flexibility refers to the ability to change real-time decision-making strategy in response to negative or positive feedback (Dajani and Uddin, 2015). In contrast, cognitive rigidity describes the resistance to making behavioral adjustments to actions that previously led to a positive outcome, but no longer do. Evidence that cognitive flexibility is affected by sleep loss was reported as early as 1988 (Horne, 1988). In recent work, reversal learning tasks have been employed to investigate this effect (Whitney et al., 2015, 2017; Honn et al., 2019; Lawrence-Sidebottom et al., 2020). The findings from these studies suggest an independent effect of SD on cognitive flexibility, although the studies were limited to reversal tasks. Notably, Whitney et al. (2015) found an association between SD-induced deficits in cognitive flexibility and decreased affective response to feedback, leading to the hypothesis that SD induced deficits in cognitive flexibility are due to *feedback blunting*, i.e., decreased feedback salience.

Animal models are important for effectively investigating the circuit-specific neurobiochemical mechanisms of reversal learning. While research involving human subjects has elucidated the importance of cortico-striatal networks for reversal learning (Fellows and Farah, 2003; Hornak et al., 2004; Remijnse et al., 2005; Ghahremani et al., 2010), experiments using animals may be more appropriate for investigating intentional perturbations of the underlying physiologic mechanisms. To this end, both primate (Smith et al., 1999; Lee et al., 2007) and rodent (Floresco et al., 2009; Klanker et al., 2013; Graybeal et al., 2014; Hurtubise and Howland, 2017; Radke et al., 2019; Bergstrom et al., 2020; Epp et al., 2021; Kipp et al., 2021; Lhost et al., 2021; Pham et al., 2022) models of cognitive flexibility have been employed and provide mechanistic explanation of post-reversal performance. However, research on the effects of SD on rodent cognitive flexibility is sparse and even contradictory (Karatsoreos et al., 2011; Walsh et al., 2011; Leenaars et al., 2012; Foakes et al., 2022). Previous research has demonstrated how SD can decrease rats’ ability to suppress action during a “no-go” stimulus (Borquez et al., 2014). In the present study we investigate this area using a relatively simple pairwise discrimination reversal task with a touchscreen apparatus (Graybeal et al., 2014; Turner et al., 2017; Dumont et al., 2021; Palmer et al., 2021). This approach has several advantages. First, the pairwise discrimination task presents preference equivalent stimuli (*viz* visual images). When stimuli are not matched with preference it can bias discrimination outcomes (Antunes and Biala, 2012; Lee et al., 2013; Foakes et al., 2022). Second, as an operant paradigm, the pairwise discrimination task utilizes appetitive instead of aversive performance incentives, which can lead to stress and adverse emotional states. Third, the pairwise discrimination task is executed in a high throughput touchscreen operant system that can easily alter the experimental parameters (Minini and Jeffery, 2006; Bussey et al., 2008; Bartko et al., 2011; McCarthy et al., 2011; Shepherd et al., 2016). For example, in the pairwise discrimination task, the implementation of correction trials, where stimulus presentation following an incorrect response is unchanged until a correct response is made, allows detection of post-reversal response stagnation. The settings and versatility of the task together with the findings of Karatsoreos et al. (2011) and Foakes et al. (2022), merits the hypothesis that SD prior to reversal will induce cognitive rigidity as evidenced by performance decrements in terms of response perseveration of previously correct responses.

## Materials and methods

### Subjects

Male Long-Evans rats aged 9–15 weeks were bred inhouse, provided enrichment and maintained on a 12/12 h light dark cycle at 24 ± 2°C with restricted access to food, but had *ad lib* access to water. Rats were housed in pairs within microisolator cages (Techniplast) containing a custom-made cage divider that allowed for smelling and nose touching their cage mate but not rough play or food sharing. The divider protected individual feeding regimens as well as limited socialization. Rats were randomly assigned to the sleep deprivation (*SD*; *n* = 6) or to the undisrupted sleep control group (CONT; *n* = 11). All procedures were reviewed and approved by the Washington State University Institutional Animal Care and Use Committee and the US Army Medical Research and Development Commands Animal Care and Use Office of Research Protections.

### Pairwise discrimination protocol

**FIGURE 1:**
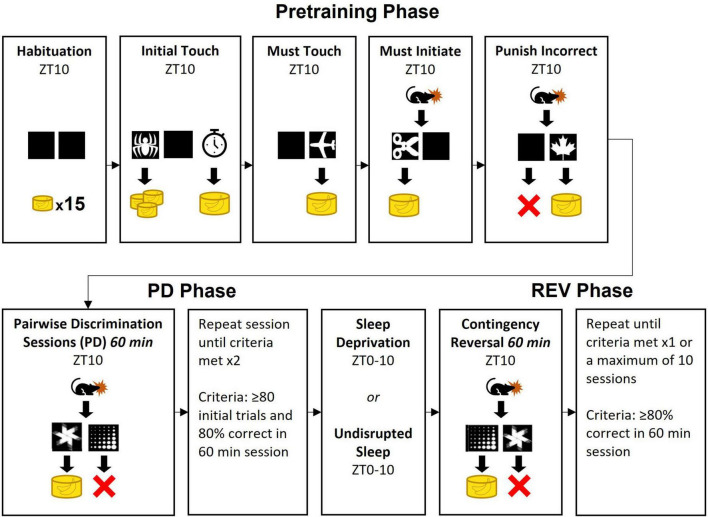
The three phases of the pairwise discrimination operant task. Images in the pretraining phase were randomly selected from the manufacturer provided image repository. Yellow cylindrical icons indicate number of banana-flavored sucrose pellets dispensed. In Initial Touch, the clock icon indicates the outcome if the rat does not touch the image within 30 s. In Must Initiate and all subsequent phases, the rat icon indicates the added requirement of the rat starting each trial manually. In Punish Incorrect and subsequent phases, the X indicates a 5 s timeout. The opposite rewarded image used for the pairwise discrimination training (PD) phase was used for the reversal (REV) phase.

The Bussey-Saksida touchscreen operant platform (Lafayette) controlled by ABETII software (version 22.03.22-touch-release-0-g236135349) was used to conduct the pairwise discrimination reversal paradigm. For both *SD* and control rats, handling and training sessions were initiated at zeitgeber time 10 (ZT10). To incentivize performance 1 week prior to habituation, rats were weighed and given 90% of the predicted food (0.7 g/100 g body weight) based on male Long Evans development curves reported by Envigo. Thereafter, rats were weighed each weekday and kept at 83–87% of the predicted body weight with feeding following session completion. Two days before the rats were scheduled to begin training, they were provided 10–15 banana-flavored sucrose pellets (45 mg; Bio-Serv) in their home cage to reduce palate neophobia. The rats were also handled four times before the beginning of training to acclimate transport in and out of the operant chambers. The training was composed of three phases: pretraining, pairwise discrimination training (PD) and reversal (REV) (Figure 1) per the manufacturer provided *Pairwise Visual Discrimination Task for Rat Touch Screen Systems and ABET II* manual (#89540Rv2-Feb 2011; Campden/Lafayette Instruments). Image placement (e.g., right or left side) was pseudo-randomly determined such that a single image did not appear on a given side more than three consecutive times. Rats in the SD and control groups experienced the same pretraining, PD, and REV protocols.

### Pretraining phase

The pretraining phase was implemented as five steps of progressively increasing complexity: Habituation, Initial Touch, Must Touch, Must Initiate, and Punish Incorrect. The Habituation and Initial Touch sessions were comprised of a single session, while Must Touch, Must Initiate, and Punish Incorrect sessions consisted of up to 5 sessions each (Figure 1).

#### Habituation and Initial Touch

During the Habituation session, the rats were provided with 15 sucrose pellets in the food tray and allowed to explore the operant chamber for 30 min. To associate the images on the screen with a food reward, in the subsequent Initial Touch session an image was presented on the left or right side. If the rats touched the image, they received three pellets. Touches on the blank side of the screen were unrewarded. After 30 s, if the rat did not touch the image, they received one pellet. Whenever pellets were dispensed, a tone was played (2 kHz, 1 s) and the tray light was turned on until the rat touched the food tray. Once the rat touched the food tray, a 20 s intertrial interval (ITI) was initiated before proceeding to the next trial. This session continued for either 60 min or 100 trials, whichever condition was met first. There was a possibility of receiving up to 300 pellets during this session (100 trials × 3 pellets/correct image) and we therefore limited this session to a single instance.

#### Must Touch

Rats then progressed to Must Touch. In this phase, trials began with an image being presented on the right or left side of the screen. The trials would not end until the rat touched the image. Upon doing so, the same tone as before was played, 1 pellet was dispensed to the food tray, and the tray light was turned on. Once the rat collected the pellet, the tray light was turned off and a 20 s ITI preceded the next trial. Daily sessions lasting 60 min were repeated until the rat completed at least 80 trials within a single session, or they completed 5 sessions of Must Touch to advance.

#### Must Initiate

This phase proceeded the same as Must Touch except with the added requirement that rats had to manually start trials. Upon completion of the 20 s ITI from a previous trial, the food tray light was turned on, and the next trial would not start until the rat touched the food tray (which also turned off the light). Criteria for stopping individual sessions and for advancing to the next step were identical to Must Touch.

#### Punish Incorrect

Finally, the rats had to incorporate previous pre-training objectives in addition to discriminating the blank side of the screen from the side displaying an image. As with Must Initiate, in this phase rats were required to start all trials via nose-poke into the food tray. Correct responses (i.e., touches on the side of the screen containing the image) produced the same outcome as in Must Initiate. However, whenever the rats touched the incorrect (blank) side the chamber light came on for 5 s, then a 20 s ITI occurred, after which the rats entered a correction trial. For correction trials, the stimulus was presented on the same side as the previous trial. Continued incorrect responses led to repeated correction trials with the image on the same side. Correct responses would break the loop of correction trials, leading to a new trial with pseudo-random placement of the image. Sessions lasted for 60 min or until 100 trials were performed, whichever condition was met first. After completing at least 80 trials with 80% correct the rats moved to the Pairwise Discrimination training phase. If rats did not achieve this after five sessions, they were still advanced to Pairwise Discrimination training.

### Pairwise discrimination training phase

At the beginning of PD, the rats were randomly assigned to either “fan” or “marbles” as the correct image. They proceeded with daily 60-min sessions initiated at ZT-10. During a session, rats were allowed to complete trials at their own pace, but a session ended early if the rat completed 100 initial trials.

*Initial trials* were initiated with nose entry into the food tray, as before. At this point, two images (“fan” and “marbles”) were displayed on the two screens in the operant chamber. If the rat selected the correct response, they received a sucrose pellet, a correct tone (2 kHz, 1 s) played, and the food tray light turned on. Once the rat retrieved the sucrose pellet, the food tray light turned off and a 20 s ITI was initiated, after which the next initial trial could be started by nose entry into the magazine.

However, if a rat selected the incorrect image during an initial trial, they entered a loop of *correction trials*. In this case, they experienced a 5 s delay with the chamber light on. The chamber light then turned off and a 20 s ITI was initiated. The rat then had to initiate the correction trial via nose-poke into the food tray (as in Must Initiate). Images were presented again in the same left/right positions as before. Incorrect responses during a correction trial resulted in another correction trial with the images presented in the same left/right positions again. Correction trials would then repeat until the rat gave a correct response. A correct response in a correction trial would break the loop and yielded the same stimuli as a correct initial trial, after which the rat could proceed to the next initial trial via nose entry into a magazine.

Rats proceeded with this phase until they reached criteria (completion of at least 80 initial trials with at least 80% correct on two separate sessions). Upon reaching criteria, rats were advanced to the REV phase. All 17 rats met criteria.

### Sleep deprivation

During the 10 h prior to the first session of the REV phase (i.e., ZT0-10), rats in the SD group were sleep deprived while the control group was left undisturbed. SD was achieved using an automated rotating bar (Pinnacle) on the cage floor, with the settings reported in Ward et al. (2017). Briefly, the bar rotated for 4 s randomly every 4–16 s, and changed rotation direction every 10–40 s. For the first 6 h the automated SD device was used alone but for the remaining 4 h of the 10 h SD period, a trained researcher was present to ensure complete SD occurred. If the researcher observed the rat attempting sleep (despite the rotating bar) they gently stroked the rats’ whiskers with an artisan’s paint brush. This hybrid approach was taken because pilot data using the rotating bar system showed that one of four rats acclimated and was able to sleep in these chambers after 6 h, consistent with other reports (Wooden et al., 2014).

### Reversal phase

**FIGURE 2:**
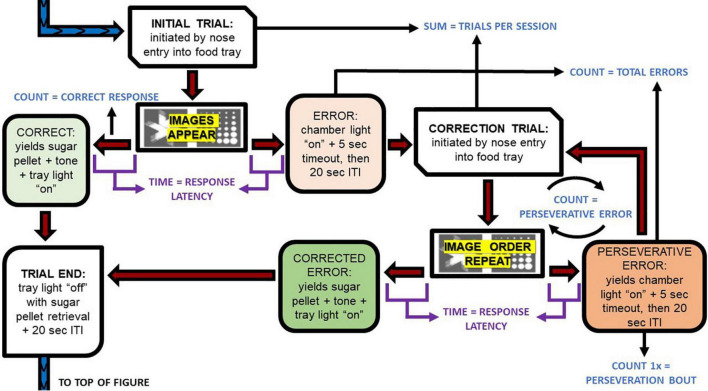
Pairwise discrimination trial progression and dependent variable extractions. This flow chart depicts the execution of the task. The blue and purple font depict the primary dependent variables described in Table 1 and how they were extracted for analyses. ITI, intertrial interval.

In the REV phase, the image that when selected previously resulted in an error (e.g., the marble image in Figures 1, 2), now resulted in a correct response and was rewarded with a sucrose pellet, while the previously rewarded image became the incorrect response. Otherwise, the procedure was kept identical to the PD Phase. Rats continued REV sessions until they met performance criterion of 80% correct trials during a single session, or until 10 sessions were completed.

### Behavioral outcome variables

Rat performance on the PD and REV phases was tracked using multiple outcome variables, including the number of sessions to criteria, number of correct responses, percent correct responses, and total errors. The rats’ capacity to complete trials in a timely manner was measured with trials per session and response latency. Additionally, the inclusion of correction trial loops in the protocol (see section “Pairwise discrimination training phase”) allowed us to measure response perseveration, viz. repetition of an erroneous response. Entries into the loop (perseveration bouts) and iterations of the loop (perseverative errors) were counted. Operational definitions are provided in Table 1.

**TABLE 1:** Operational definitions of the experiment.

Initial trials	Trials that (1) was the first trial of the session, or (2) occurred after a correct response. In initial trials, the right/left placement of the two images was pseudo-random and independent from the previous trial.
Correction trials	Trials occurring after an error. In these trials, the right/left placement of the two images was repeated from the previous trial.
Trials per session	The total number of all trials (initial trials plus correction trials).
Response latency	The time between the display of the images and the screen touch (i.e., rat response) during both initial and correction trials.
Correct responses	Number of correct responses given during initial trials. Does not include correct responses given during correction trials.
Percent correct responses	Correct responses divided by initial trials.
Total errors	Total number of incorrect responses given during initial trials and correction trials.
Perseverative errors	Total number of incorrect responses given during correction trials.
Perseveration bouts	Number of times that a continuous string of 1 or more perseverative errors occurred. This was calculated by counting the number of incorrect responses given during the first correction trial following a failed initial trial. For example, if a rat answers incorrectly during an initial trial, and then answers incorrectly again during the subsequent correction trial, perseveration bouts would increment. However, after this, repeated failed correction trials for the same initial trial would not increment perseveration bouts. Perseveration bouts would only increment again if the rat responded correctly to break the correction loop, and then answered incorrectly on a different initial trial and the following correction trial.

### Data analyses

Once a rat reached criteria during the PD or REV phases, they discontinued future sessions in that phase and their final session metric carried forward. For trials per session, response latencies, correct responses, percent correct responses, total errors, perseverative errors, and perseveration bouts IBM SPSS version 28.0 statistics package performed mixed 2-way ANOVAS on the within factor of sessions (1–10) and the between factor of treatment (SD vs. CONT) for the PD and REV phases independently. Studentized *t*-tests were used to compare the number of sessions to meet criteria in SD and CONT rats, along with pairwise comparisons of each session when appropriate. Alpha levels for statistical significance were set to *p* < 0.05, while *p* ≥ 0.05 and ≤ 0.07 was interpreted as a non-significant trend.

## Results

As an index of task acquisition and performance efficiencies, the main effect of sessions was statistically significant for all outcome variables and is not mentioned hereafter, instead we focus on the interaction of treatment and sessions. The results are presented in metrics of general performance, correct responses, and errors.

### Sessions to criteria, trials per session, and response latencies

**FIGURE 3:**
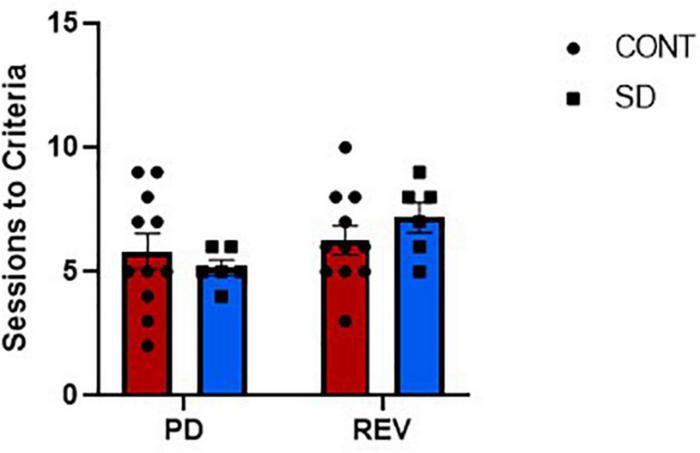
The sessions to reach performance criteria for both the control group (CONT) and the sleep deprivation group (SD) were similar in the pairwise discrimination training (PD) and the reversal (REV) phases. Data are reported in means and standard errors.

**FIGURE 4:**
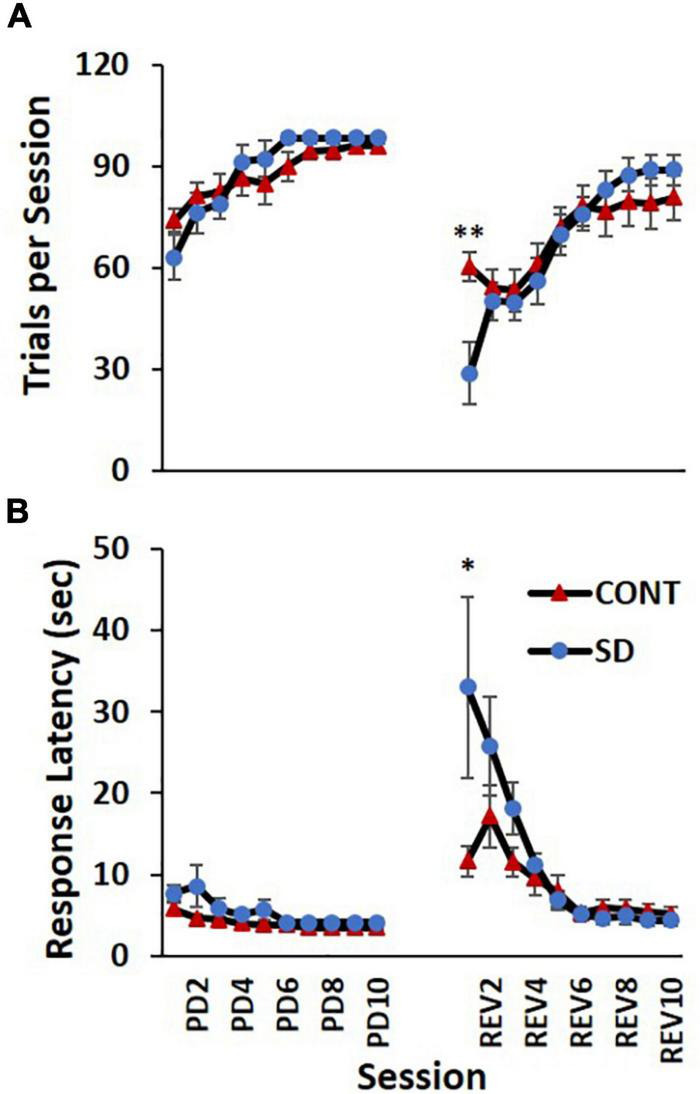
Performance decrements immediately following sleep deprivation (SD). Both the control (CONT) and SD groups had comparable performance on the number of trials per session **(A)** and response latency **(B)** during the pairwise discrimination training (PD) phase. The SD group had a decreased capacity to complete trials during the initial reversal (REV) phase as indicated by fewer trials per session and increased response latency. Data are reported in means and standard errors (*p < 0.05, **p < 0.01).

Rats in the SD group took an average 0.6 less sessions and an additional 0.9 more sessions to reach performance criteria compared to CONT, in the PD and REV phases, respectively (Figure 3). However, this difference was not statistically significant. The increasing number of trials per session were comparable between SD and CONT groups during the PD phase, but varied during the REV phase [*F*_(9, 135)_ = 3.79, *p* < 0.001; Figure 4A]. Specifically, in first REV session, CONT rats had an average of 32 more trials than SD rats (*p* = 0.005). Likewise, response latencies were similar between groups during the PD phase, but during the early REV phase response times were delayed in the SD group [*F*_(9, 135)_ = 3.91, *p* < 0.001; Figure 4B]. Despite increased variability in rats subjected to SD, pairwise comparisons of the first REV session found response latencies on average 21.3 s longer in those rats compared to controls (*p* = 0.03). These performance decrements rapidly diminished within the first few sessions; by REV session 4 the SD rats had average response latencies only 1.7 s greater than controls. In sum, both treatment groups tended toward more trials per session and lower response latencies as the REV phase progressed, but the expected performance decrements incurred by reversing the response contingencies was exacerbated by SD.

### Correct responses

**FIGURE 5:**
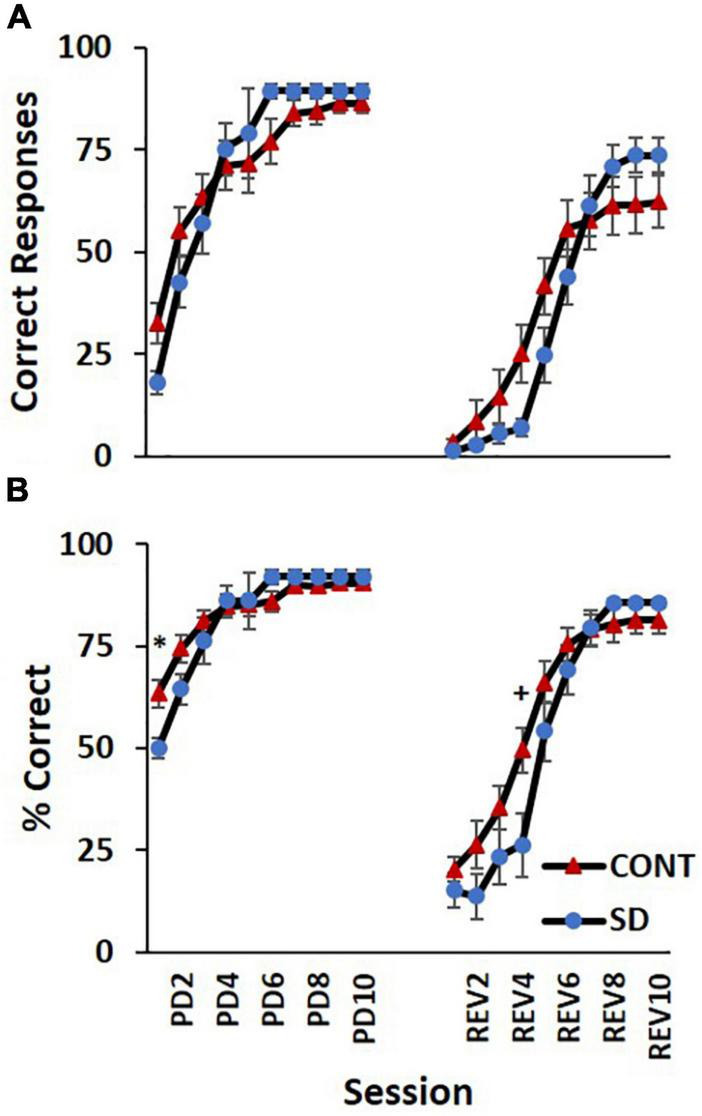
Correct responses in the pairwise discrimination (PD) and reversal (REV) phases are altered in the sleep deprivation (SD) group. Group differences were observed in the PD phase for both measures with SD rats performing worse initially, but better toward the end. Compared to the control group (CONT), the SD group performed worse in correct responses **(A)** and percent correct **(B)** during the initial sessions of the reversal phase. By the end of the reversal phase, the SD group began to surpass the control group. Data are reported in means and standard errors (^+^*p* < 0.07, *p < 0.05).

Correct responses (Figure 5A) increased across sessions in both the PD and REV phases. Regardless of receiving the same experimental conditions, by the end of the PD phase, SD rats had more correct responses on average than CONT rats [*F*_(9, 135)_ = 2.15, *p* = 0.029], however, statistically significant session-by-session comparisons were not detected in *post hoc* analyses. Similarly, in the REV phase, SD rats had fewer correct response than CONT rats and then surpassed them at the latter sessions [*F*_(9, 135)_ = 2.48, *p* = 0.012], but as with the PD phase, statistically significant session-by-session comparisons were not detected. Group differences in the percent of correct responses were also observed in the PD [*F*_(9, 135)_ = 3.18, *p* = 0.002; Figure 5B] and REV phases [*F*_(9, 135)_ = 2.25, *p* = 0.023]. In PD session 1, CONT rats had 13 more percent correct responses than SD rats (*p* = 0.037), but this effect diminished throughout PD; on PD sessions 7, 8, 9, and 10 performance of the SD and CONT groups was very similar, with the SD group demonstrating only 1–2 more percent correct responses than the CONT group. A decelerated pattern of percent correct responses manifested following SD such that on REV session 4, the difference between the two groups approached significance with SD rats demonstrating 23 less percent correct responses than rats with undisrupted sleep (*p* = 0.051). Thus, even when correct responses are taken as a percentage of initial trials, i.e., percent correct responses, SD rats still underperformed in comparison to CONT rats early in the REV phase. This pattern appeared to invert toward the end of the REV phase, with the SD rats performing better on initial trials than the controls, although the effect was not statistically significant. For example, SD rats trended with an average of 16, 20 and 18% more correct responses and an average of 7, 5 and 5% more percent correct responses than CONT rats on REV sessions 8–10.

### Errors

**FIGURE 6:**
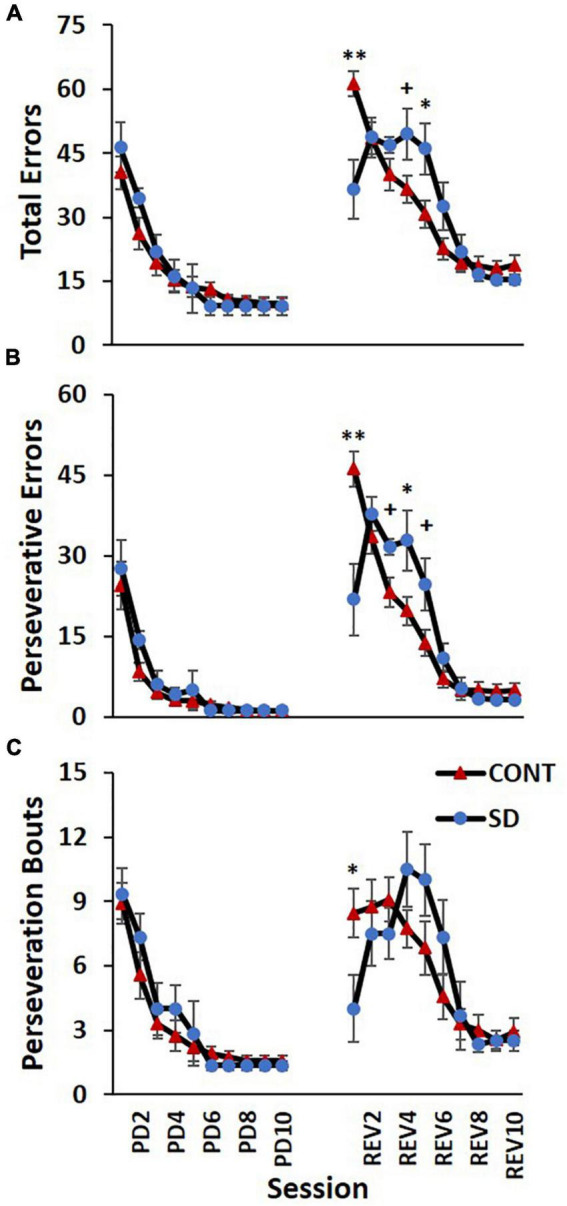
Sleep deprivation-induced increases in perseveration and performance decrements after image reversal. The control (CONT) and sleep deprivation (SD) groups performed similarly on all three measures during the PD phase. Initially, the SD group had fewer total errors **(A)**, perseverative errors **(B)**, and perseveration bouts **(C)** than the CONT group at the start of the reversal phase (REV). However, during the middle of the reversal phase the SD group performed worse than the CONT group. During the latter half of the pairwise discrimination training phase (PD), the SD group produced fewer total errors than the CONT group. Data are reported in means and standard errors (^+^*p* < 0.07, *p < 0.05, **p < 0.01).

In the REV phase, statistically significant changes between treatment groups in total errors were also observed [*F*_(9, 135)_ = 6.55, *p* < 0.001; Figure 6A], although the direction of the changes varied during the REV phase. In the first REV session, SD rats produced 24.9 less errors than CONT rats (*p* = 0.0025). On REV session 4 the directionality of the changes switched, with SD rats now producing 13.0 more errors; this difference on REV session 4 approached significance on *post hoc* analyses (*p* = 0.069). Additionally, on REV session 5, SD rats had 15.3 more total errors compared to CONT rats (*p* = 0.038).

The frequency of perseverative errors followed a similar trend and was robustly affected in the REV phase signifying the detrimental effects of SD on cognitive rigidity [*F*_(9, 135)_ = 7.93, *p* < 0.001; Figure 6B]. As with total errors, the directionality of the differences varied during the REV phase. On the first REV session, the SD group had 24.4 less perseverative errors than the CONT group (*p* = 0.004), but this was followed by a mid-REV phase performance decrement in SD rats—on REV sessions 3-5, SD rats produced 37% (*p* = 0.063), 66% (*p* = 0.043), and 80% (*p* = 0.054) more perseverative errors than CONT rats.

This pattern—the SD group displaying a decrease in errors on the first REV session, followed by a marked increase in errors mid-way through the REV phase—was also observed with perseveration bouts [*F*_(9, 135)_ = 2.43, *p* = 0.014; Figure 6C] with the SD group producing 4.5 less perseveration bouts than the CONT group on session 1 of REV (*p* = 0.044). There were also 36, 47, and 61% increases in perseveration bouts in SD rats relative to CONT rats on sessions 4–6, but the pairwise comparisons were not statistically significant. Taken together with the findings for correct responses, this solidifies the pattern of compromised REV phase performance for the rats following SD.

## Discussion

Overall, our hypothesis that sleep loss confers cognitive rigidity was supported. This is consistent with other findings from animal (Karatsoreos et al., 2011; Foakes et al., 2022) and human research (Whitney et al., 2015, 2017; Honn et al., 2019; Lawrence-Sidebottom et al., 2020). Our findings also show that the touchscreen operant platform and the relatively simple pairwise discrimination reversal paradigm is appropriate for characterizing the effects of SD on adaptive decision making. The incorporation of correction trials in the PD paradigm allows insight into incorrect responses and maladaptive response stagnation.

We report that the immediate effects of SD on generalized fatigue were apparent in REV performance. Although the definition of fatigue is nebulous (Whitney and Hinson, 2010), herein it is operationally defined as a decreased capacity to complete trials in a timely manner (regardless of motivational, motoric or vigilance origin). Our proxy variables for fatigue were response latency and total trials. They index trial and session rates of response capacity, respectively, and were inversely correlated (Figure 4). Both variables indicate exacerbated fatigue in SD rats during the initial session of the REV phase with half the trials and three times the latency to respond. This is consistent with previous reports indicating the negative effects SD on vigilance and attention (Banks and Dinges, 2007; Goel et al., 2009; Killgore, 2010; Kayser et al., 2022).

**FIGURE 7:**
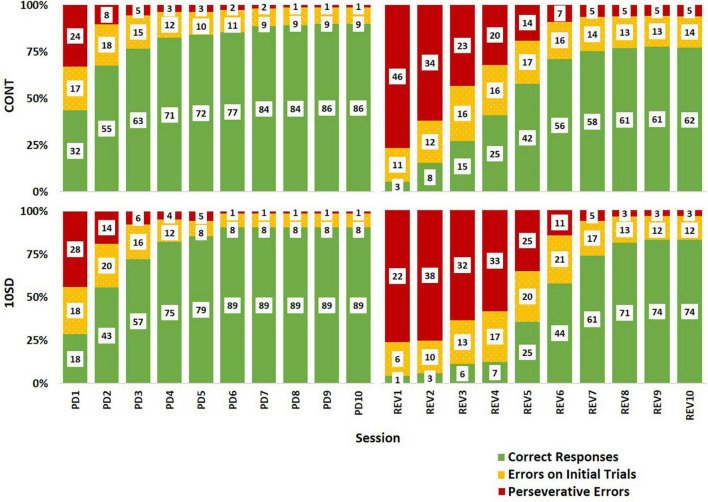
Response type distributions depicting sleep deprivation-induced cognitive rigidity as by evidenced by decelerated adaptive responses in sleep deprived rats following reversal. The control group (CONT) and sleep deprivation group (SD) had similar distributions of response types during the pairwise discrimination training (PD) phase. Both groups had a high proportion of perseverative errors (red) at the beginning of the reversal (REV) phase, but the SD group had a higher proportion of perseverative errors for more sessions. This trend was also observed in the errors on initial trials (yellow) accompanied by fewer correct responses (green). Inset values represent the mean number of responses by type. The height of bars indicates the proportionality of response types relative to each other (summed bar height = 100%).

The paucity of responding subsequent to SD could be misconstrued as better performance in SD rats when errors on the first REV session are examined. To clarify this, we probed the relative frequencies of correct responses, errors made during initial trials, and errors made during correction trials (i.e., perseverative errors) per each session (Figure 7). Graphing the outcomes proportionally to each other indicates that the SD rats manifest decreased accuracy and elevated perseveration similar to the control rats at the beginning of REV. However, SD rat performance is characterized by a decelerated adaptive response, as determined by increased perseveration for a protracted period and taking more sessions to elicit improved performance. Thus, the effect on cognitive rigidity remained well after the recovery sleep opportunity and dissipated fatigue (Figure 4). The reported performance deficits appeared both in terms of generally decreased ability to adapt to changed contingencies (e.g., mid-REV session CONT rats averaged about twice the percent correct responses as CONT rats), and also in terms of perseveration on an incorrect response even after multiple unaltered stimulus presentations and unrewarded responses (e.g., mid-REV session SD rats averaged two thirds more perseverative errors than CONT rats).

A possible explanation for these findings is that SD-induced fatigue at the beginning of REV impeded SD rats’ learning during the first REV session; thus, SD rats did not begin learning the reversed contingencies until the second session. A SD-induced one-session delay in adaptation to reversal is a plausible interpretation of Figure 7. Such an interpretation would support the role of impaired memory binding (i.e., within the rats’ brains, impaired linking of image choice to observed outcome; Zimmer et al., 2012) as an important causal factor for the observed effects of SD on cognitive flexibility. Indeed, there is ample evidence of SD’s detrimental effect on cognitive tasks that require memory binding (Harrison and Horne, 1999; Killgore et al., 2006; Yoo et al., 2007b; Chai et al., 2020; Kurinec et al., 2021). In theory, intact memory binding would be important for the initial association of choices to outcomes, as well as forming new replacement associations when contingencies change. Impaired memory binding on the first day of REV would effectively prevent the SD rats from internalizing the changed contingencies of the task. Thus, rats would not start developing new associations between their choices and the outcomes until the second day. Such an interpretation could also explain feedback blunting in sleep-deprived human subjects performing reversal tasks (Whitney et al., 2015): impaired memory binding may have decreased the subjects’ affective response to feedback—in essence, the subjects may have been less able to associate their choices with observed outcomes and were less prone to affective responses when the outcomes occurred. Some aspects of Whitney et al. (2015) findings support this interpretation; in their 2015 study, pre-task total SD impaired initial task performance, reversal task performance, and positive effect of practice. Each of these outcome metrics rely on sufficient memory binding; therefore the impairments reported in the 2015 study could be explained by diminished memory binding following SD.

Another explanation for our current findings is that SD rats were unable to learn during the first REV session because they did not complete enough trials due to fatigue. This explanation is unlikely since it assumes a minimum number of trials that must be achieved that precedes the ability to associate responses with outcomes. SD rats completed half the number of trials as CONT rats on the first REV session but exhibited essentially no learning between the first and second REV sessions (Figure 7). Therefore, it is more likely that the relationship between number of trials completed, and internalization of feedback was masked by a separate process such as impaired memory binding on the first REV session.

Of note, the results of the present study—a pairwise discrimination reversal task—differ from those of a similarly appetitive operant switch task (Leenaars et al., 2012), in which SD prior to reversal was not found to impair reversal learning. This may be because the pairwise discrimination reversal task is more complex, relying on interpretation of visual stimuli instead of spatial position. The apparent discrepancy highlights the importance of using multiple paradigms to measure cognitive flexibility—some paradigms may demonstrate an effect while others may not. Another explanation for the discrepancy between our results and the operant switch task results is the rat strain tested (Long Evans vs. Wistar rats).

There are some potential limitations to the current study. To begin with, the current study uses schedule of reinforcement that is a fixed ratio of one to one (every correct response resulted in reward delivery), whereas studies of reversal tasks in humans typically use probabilistic and leaner reinforcement schedules (Izquierdo et al., 2017). Additionally, reversal tasks are not the only method for assessing cognitive flexibility. Others include intradimensional set-shifting tasks, extradimensional set-shifting tasks, and strategy shifting tasks (McCoy et al., 2007; Klanker et al., 2013). Therefore, although the cognitive demands in this study were sufficient to produce an SD effect on performance, the deterministic nature and simplicity of the pairwise discrimination reversal task may limit its translatability. Still, the reductionistic assay may prove more conducive to the discrete identification of neurocircuits. Another caveat is the detection of statistically significant group differences during the PD phase for correct responses and percent correct responses. All rats received similar treatment, regardless of group, throughout the PD phase and these findings were unexpected. The direction of the changes suggest that they do not detract from the conclusions of this study, in that they likely deadened the reported effects as SD rats had more correct responses going into the REV phase; any observed SD-induced deficits would need to have overcome that potential advantage. It would be more difficult to interpret had the CONT rats exhibited better performance before the REV phase.

Despite these limitations, the current results demonstrate the effectiveness of using a pairwise discrimination reversal task to elicit the effect of sleep loss on cognitive flexibility in rats, distinct from fatigue. This adds an additional simple, high-throughput behavioral assay that can be used for investigating the effect of SD on rodent cognitive flexibility. The present study was executed using wild type rats and did not equip or instrument rats, but the paradigm could be useful in elucidating the mechanisms underpinning reversal learning performance decrements from insufficient sleep and the role of neurotransmitters and brain networks via drugs, mutant rat models, opto- or pharmacogenetic research strategies (Klanker et al., 2013; Radke et al., 2019; Bergstrom et al., 2020; Lhost et al., 2021). Identification of such mechanisms will inform treatment strategies on how to overcome society’s growing problem of sleep insufficiency.

## Data availability statement

The raw data supporting the conclusions of this article will be made available by the authors, without undue reservation.

## Ethics statement

This animal study was reviewed and approved by the Washington State University Institutional Animal Care and Use Committee and the United States Army Medical Research and Development Commands Animal Care and Use Office of Research Protections.

## Author contributions

BS: data curation, validation, formal analysis, manuscript writing—original draft, and review and editing. MS: resources, methodology, investigation, manuscript review and editing, and project administration. DH: methodology, investigation, data curation, and manuscript review and editing. CD: conceptualization, formal analysis, manuscript writing—original draft, and review and editing, validation, visualization, supervision, and funding acquisition. All authors contributed to the article and approved the submitted version.
