# Corrigendum: Image discrimination reversal learning is impaired by sleep deprivation in rats: Cognitive rigidity or fatigue?

**DOI:** 10.3389/fnsys.2023.1141071

**Published:** 2023-01-25

**Authors:** Brian K. Strobel, Michelle A. Schmidt, Daniel O. Harvey, Christopher J. Davis

**Affiliations:** Department of Translational Medicine and Physiology, Sleep and Performance Research Center, Elson S. Floyd College of Medicine, Steve Gleason Institute for Neuroscience, Washington State University, Spokane, WA, United States

**Keywords:** pairwise discrimination, cognitive flexibility, operant conditioning, adaptive decision feedback, perseverative errors, response latency

In the published article, there was an error in the Funding statement. The correct Funding statement appears below.

This work was funded by a USAMRDC W81XWH-18-1-0100 award. The funders had no role in study design, data collection and analysis, decision to publish, or manuscript preparation.

The authors apologize for this error and state that this does not change the scientific conclusions of the article in any way. The original article has been updated.

